# On the Cure of Eruptions of the Head and Face in Children*Medico-Chirurgical Review.

**Published:** 1846-10

**Authors:** 


### PART VI.—SELECTIONS.

1. *On the Cure of Eruptions of the Head and Face in Children.**—[M. Trousseau makes some interesting remarks, in his *Journal de Medecine*, upon the rules that should guide the practitioner in endeavoring to heal the eruptions, sores, &c., which affect the head and face of young children. To avoid circumlocution, we will employ, in the extracts we make from the paper, the term by which these are designated in France —*les gourmes*—equivalent to our appellation "breakings out."]

It is a popular opinion that danger attends the attempt to heal these, and this is sometimes true when their manifestation is connected with a morbid diathesis. Others, however, unconnected with this, do much mischief, and should be healed at once. A diathesis may be acquired or congenital; and the *suppurative diathesis* is that, which of all others, is most evidently acquired. The "*gourmes*" are, indeed, generally one of the manifestations of this; while in other cases the *dartrous* diathesis, which is usually hereditary, plays an important part in generating the eruption. The form of the "*gourmes*" will vary, according as one or the other of these prevail. Impetigo, ecthyma, impetiginous eczema, intertrigo, furunculus, superficial phlegmon, and opthalmia, are more especially connected with the suppurative diathesis; while lichen, psoriasis, eczema rubrum, pityriasis favus, and chronic inflammation of the eyelid, are more often dependant upon the dartrous diathesis.

"1. When, from distress, neglect, or other cause, a superficial phlegmasia becomes in the course of several months converted into a suppurating sore, in the groin, behind the ears, or upon the scalp of the child, the economy, which at first suffered from the presence of an useless discharge, accustoms itself to it to such an extent that, although its suppression at an early period would have been very advantageous, this must now be accomplished cautiously, or disease and ill health will result. 2. Again, when an impetigo suddenly develops itself in a child previously in ill health, and becomes chronic, the health may become manifestly improved as long as the eruption continues. It is evident that, for a certain period at least, it should not be meddled with, and even then that its cure should be very cautiously undertaken. 3. The development of the "*gourmes*" may be the signal of serious disorders in a child prior to this in good health. In this case, their cure, if fever be present, should be set about at once, without any fear of the pretended effects of a retrocession. 4. When a child's health is good, we must endeavor by every means to prevent the establishment of the "*gourmes;*"

* Medico-Chirurgical Review.

for, if suppuration be accidently established, it may give rise to other suppurations—in fact, generate a suppurative diathesis. This diathesis again may manifest itself, not only on the skin and mucous membranes, but also in the internal organs; and thus, in children suffering from "*gourmes*," variola, rubeola, scarlatina, &c., are always more fatal. 5. When the "*gourmes*" invade important parts, as the eyes, nasal fossæ, auditory canal, &c., we must use every means to prevent their extension.

"*Treatment.*—The superficial *excoriations* which are found behind the ears and between the folds of the skin in gross children, usually arise from negligence, and often disappear upon the mere observance of cleanliness. Soapy baths, dusting them with lycopodium, or the interposition of lint moistened in olive oil, usually suffice to dry them up; but when they are obstinate, white precipitate ointment, (ʒj. ad ʒx. axung,) or Galen's cerate, may be employed. Frequently, to cure the intertrigo behind the ears, it suffices to take care that the string of the cap be not too tightly tied, or to prevent the surfaces of the skin from coming in contact with each other.

*Impetigo*, *impetiginous eczema*, and *ecthyma* in their *acute* form require special treatment. Dr. Trousseau, regarding the two first as true eruptive fevers, just as scarlatina, variola, &c., is careful in not suppressing them too rapidly, although he does not encourage their development. So far from this, believing with Sydenham, that our object should be to prevent eruptive diseases becoming confluent, he prescribes prolonged baths, abstinence, acid drinks, and mild laxatives. The children are not to be too much covered up nor to be kept in bed. Excessive cleanliness is to be observed, and great care taken that they do not scratch the pustules, and diffuse the disease with their nails over other portions of the body. When the febrile action has ceased, we have to do with a mere local disease, and must get rid of it as soon as possible. Unfortunately, however, impetigo oftentimes succeeds to measles and scarlatina; in which case, our proceedings must be more circumspect. If the impetigo be too rapidly healed, in this case the lungs, or some other internal organ, will very probably become diseased, we having thus destroyed the revulsive affection of the skin which acted as a preventive, or as a curative if they were already affected. There are circumstances, however, in which such caution would be misplaced. Thus a violent inflammation of the ocular mucous membrane may spread to the eye itself, or a very severe eczema behind the ear may give rise to dangerous or even fatal enlargement of the cervical glands. In both these cases we must at once cure the eruption, as it gives rise to greater evils than we have reason to fear from its repercussion.

"When the *impetigo* and *eczema* become chronic, and the condition of no internal organ causes alarm, I treat them with baths, ointments, lotions, purgatives, blisters, or depuratives. *Alkaline baths* are the best of remedies when the disease is attended with itching. To 75 or 100 quarts of water I usually add from 12 to 20 drachms of sub-carbonate of soda or potass. These baths most effectually clean the skin, soften the crusts, and relieve the pruritus. The dreadful suffering this last causes proves its relief alone is no slight advantage. With a solution rather stronger than that employed for the baths, lotions may be made and locally applied two or three times daily. These baths are suitable for the *dry forms of eczema*, for *lichen*, and for *pityriasis*. But when the *eczema* is very *acute*, and is accompanied by great redness and abundant discharge, *mercurial baths* are to be preferred. I prepare these by adding to 50 or 70 quarts of water three or four scruples of corrosive sublimate, dissolved in 1 oz. or 1½ oz. of alcohol. I have used these baths for fourteen years in every variety of dartrous affection of the skin, with the greatest advantage. Some practitioners consider them dangerous, but I order about a thousand annually, and even for women in the weakest state and children of the earliest age without ever seeing any accidents result from their employment. I have had children placed in these baths, half the skin of whose bodies had been destroyed by eczema, and no injurious absorption of the mercury has taken place, while the epidermis has become regenerated in a few days. Very young infants should not be kept in the bath more than quarter of an hour at the farthest, but those who are more than a year old may be retained in for half an hour. The severest forms of *eczema*, *lichen*, *erythema*, and *impetiginous eczema* soon yield to these baths, and they form the most appropriate treatment of the *syphilides* of infancy. In simple, chronic *impetigo*, I find *sulphureous* baths, formed of 1 or 2 drachms of sulphuret of potash to 50 or 70 quarts of water, best. But they are especially indicated in children covered with furunculi or little sub-cutaneous abscesses. The action of these baths is no doubt chiefly topical, for ointments composed of the same materials and applied to circumscribed spots are as useful; but when we find the alkaline baths correcting acid urine, and the mercurial baths relieving syphilis, it is evident that some portion of their material is absorbed, as is also shown by the odour which the sulphureous baths impart to the secretions. Indeed, experience has proved the efficacy of alkalies and mercurials, taken internally, in moderating the dartrous diathesis, which manifests itself in herpetic eruption.

When the affections of the skin are very limited, *lotions*, composed of the same materials, in larger proportions than in the baths, may be substituted. The strength of these must depend upon the susceptibility of the skin, and condition of the lesion; but the practitioner must not be afraid of using them pretty strong, as the temporary irritation they excite is often advantageous to the affection. In the treatment of "*gourmes*" of the hairy scalp, the sulphuret of potassium may be employed in such strong solutions as to be almost caustic. The *temperature* of these lotions should be as *high* as can possibly be borne. This may seem strange advice at first, but doubtless much of the efficacy of the vapor bath in cutaneous affections depends upon the great heat thus produced, and the success attendant upon the employment of infusions of simple herbs by empirics in like manner results from their using these very hot.

"Among the *ointments*, those containing *mercury* occupy the very first place. White precipitate and calomel are usually to be preferred to red precipitate; but nothing absolute can be stated, for in apparently identical affections, sometimes the one and sometimes the other preparation proves most efficacious. The two former may be used in the proportion of one part to five or ten of cerate, and the red precipitate half as strong. In some children, lard, and in others cerate, forms the best vehicle. In some diseases of the hairy scalp, alkaline or sulphureous ointments are preferable to the mercurial ones, and this is the case especially in the moist and scabby forms. In the dry and squamous forms, ointments formed of mercury, of pitch, or of sulphate of copper, are highly useful. But I cannot too often repeat, that we must try various means, and neither allow ourselves to be too much encouraged by former success, or discouraged if we find a remedy useful in some cases of no avail in others. Even for the same disease, the practitioner should always be provided with a certain variety of remedies, which will all, some day or other, be required.

"I now come to the consideration of the employment of *blisters*. And first, let it be observed that a substance, such as Burgundy pitch, croton oil, or mercurial ointment, which, when applied, sometimes gives rise to the production of a local crop of vesicles, occasionally also leads to a *general* eczema, first acute and then chronic. This is a rare occurrence in men, rather more common in women, and very frequent in children. A few months seldom pass without my seeing, in hospital or private practice, an acute, simple or impetiginous eczema attack children, after the unavoidable employment of a temporary blister in pneumonia. Generally the disease assumes a chronic character; and if we consider that, up to this time, the child was not the subject of any cutaneous affection, we must admit the blister has been at least the occasional cause of its production. Seeing, then, that in a healthy skin a blister may develop a chronic cutaneous affection, ought we to attach much importance to this means for the treatment of

"*gourmes*," and rather ought we not reject it in the majority of cases? I have now in my wards a young child who, when the subject of a slight lichen upon some few points of the skin was ordered a blister by its attendant. A few days after, the arm to which this had been applied was covered with eczema which quickly spread over the rest of the body. I have frequently, in obedience to routine or theory, applied blisters to children affected with "*gourmes*," but have often repented doing so, and seldom seen benefit result. Believing, then, blisters only cause additional irritation without relieving that already existing, I prescribe them in cutaneous affections; but I employ them in treating the "*gourmes*" of the mucous membranes. Experience has often shown me disease behind the ear, or of the hairy scalp, alternating with opthalmia or chronic eczema of the nasal fossæ, as if the two affections were incompatible. In this case, a blister to the arm is generally useful, although sometimes the derivation will not establish itself in the direction chosen by the attendant, but obstinately tends towards its original route. We may leave the blister on the arm, at the same time endeavoring to encourage the fluxion where it seems most willingly and beneficially inclined to place itself. But if blisters are of use in the cure of these, so to say, *alternating* "*gourmes*," they are not so in "*gourmes*" resulting from *propagation*. Thus, we may often seen an impetiginous eczema gradually invade the forehead, eye-lids, conjunctiva, the rest of the face, and penetrate into the nose. I call this *propagation*, and in such a case blisters are of no avail. But if an opthalmia replaces the eczema of the skin, which in its turn acquires predominance when the opthalmia is relieved, I call it *alternating* or *compensating*, and here blisters are, in general, useful. If they are useful here, they are imperiously demanded when a bronchitis, an enteritis, a pulmonary, or intestinal catarrh is set up, and alternates with the cutaneous "*gourmes;*" for all these are but other manifestations of the same diathesis which a true pathologist must never overlook.

"To decide upon the exhibition of *purgatives* is also somewhat difficult. The popular idea is, that these medicines constitute our sheet-anchor in treating "*gourmes*." If a somewhat severe diarrhœa occurs in a child subject to these affections, we observe on the very first day the eruption becomes paler, and if it continue, the inflammatory fluxion entirely disappears, and the cure may be effected without any topical remedy. If, however, the diarrhœa is naturally, or under the influence of medicine, arrested, you find the cutaneous affection almost immediately take on all the marks of activity it had lost. So that the antagonism between the skin and the gastro-intestinal mucous membrane is evident enough. With some practitioners, an artificial and spontaneous diarrhœa are the same things—in both, there is an intestinal flux. But the observer sees things differently. In spontaneous diarrhœa all the economy is prepared for this new fluxionary movement, and when it is established, it draws within its sphere of action a multitude of secondary vital acts. In artificial diarrhœa the economy resists the cause provoking it. There is doubtless a flux from the intestinal canal established; but it is isolated, all other acts of the economy retaining their independence. Compare the condition of the man who becomes the subject of a diarrhœa, with his who takes a bottle of Seidlitz water, observe the exhaustion and *malaise* of the one, and the little inconvenience which a much greater number of stools causes to the other. A woman has not her menstrual discharge or a man his hæmorrhoidal flux at their usual period, will the taking away a far larger quantity of blood than that usually lost from the vulva of the one, or the anus of the other, have the same effect on the economy? Some persons are affected several times in a year with an erysipelatous swelling of the nose or ear; substitute for such spontaneous irritation that produced by a large blister, and see if the effect will be the same. In a spontaneous act there is such a condition of the economy, that every function is in some measure subordinate to the actions about to take place, which can hardly ever be the case when the effect is sought to be produced by a therapeutical agent, unless, indeed, the indication has been well prepared and skilfully seized.

"I have said enough to show that we must not judge of the influence which a purgative will exert by that which a spontaneous diarrhœa produces. But, if in lieu of the transitory action of a purgative given from time to time, we produce an effect from day to day, or almost continuously; or again, if a temporary action be very energetic, and frequently renewed, we may produce results less marked, it is true, than those proceeding from spontaneous diarrhœa, but yet considerable enough to be of great importance to the practitioner. It remains to inquire whether a plan so acted upon is applicable to ordinary cases? I reply, it is not. It is dangerous for young infants, whether they are at the breast, or have been weaned. Gastro-intestinal phlegmasiæ, at this age, are of a grave character, whether considered as preventive of the active nutrition so requisite at this period of life, the acute, and often fatal affections they gave rise to, or the chronic ailments they predispose to. Purgatives, to be of service in "*gourmes*," must be active, and it is easy to give rise to greater disorders than those we are seeking to combat. Such precautions are not required for adults, adolescents, or even for children above their third year, in whom these gastro-intestinal phlegmasiæ are established with difficulty, usually exempt from danger, and easily curable. If, in an infant, a slight diarrhœa, which had caused neither exhaustion nor wasting, and yet has much improved the condition of the "*gourmes*," becomes arrested, we must endeavor by the aid of purgatives, to reproduce it, and maintain it as nearly as possible in the same state it had previously existed in.

"Various vegetable ptisans have acquired a reputation as *depuratives*, and many of these, as bitter-sweet or wild pansy, and also chicory-juice, are very useful adjuvants when taken for a long time by the children who have passed their first infancy. But I must protest against the employment of *cod's-liver oil* and *hydriodate of potass* to this end, even when the "*gourmes*" can be traced to a scrofulous origin. I have almost always found these two therapeutical agents produce vesicular and papular eruptions; and, during the treatment of rickets, I have frequently been obliged to suspend the administration of cod's-liver oil, because the skin had become covered with eruptions, sufficient in many cases to excite considerable febrile action."—*Bulletin of Medical Science.*

